# Daily variations in ambulance calls for selected causes in Arkhangelsk, Russia: potential role of excessive alcohol consumption on weekends

**DOI:** 10.3402/ijch.v71i0.19124

**Published:** 2012-11-01

**Authors:** Sergei N. Drachev, Tatiana N. Unguryanu, Andrej M. Grjibovski

**Affiliations:** 1 International School of Public Health, Northern State Medical University, Arkhangelsk, Russia; 2 Department of International Public Health, Norwegian Institute of Public Health, Oslo, Norway; 3 Institute of Community Medicine, University of Tromsø, Tromsø, Norway

**Keywords:** alcohol, ambulance, binge drinking, cardiovascular, external causes, Russia

## Abstract

**Objective:**

To assess daily variations in ambulance calls for cardiovascular diseases (CVDs), mental and behavioral disorders, and external causes in Arkhangelsk, Northwest Russia, in 2000–2008.

**Study design:**

A population-based study.

**Methods:**

Data about all ambulance calls during the years 2000–2008 were obtained from the Arkhangelsk ambulance station. Information about patient's gender, age, doctor's diagnosis according to *International Classification of Diseases*, 10th revision, and the date of call were recorded. Pearson's Chi-squared tests were used for comparing proportions of ambulance calls across the week for CVDs (I00-99), mental and behavioral disorders (F00-F99), and external causes (S00-T98, V01-Y98). The ratio of incidence of ambulance calls on Saturday, Sunday, and Monday versus the rest of week was also calculated.

**Results:**

There is a significant daily variation (p < 0.001) in calls for CVDs in men and women aged 18–59 and women aged 60 years and older, with increased numbers of calls on weekends and Mondays varying between 2 and 3% excess calls. For mental and behavioral disorders, a similar pattern was found in the age group of 18–59 year-olds. Ratios for the number of calls during weekends and Mondays vs. the rest of the week were 1.05 (95% CI: 1.02–1.08) among women and 1.02 (95% CI: 1.00–1.05) among men. For external causes, a significant variation and an increase in ambulance calls during Saturdays, Sundays, and Mondays from 4 to 17% excess calls was observed for both age and gender groups.

**Conclusions:**

The observed daily variations in ambulance calls with an increased number of calls on weekends and Mondays for CVDs, mental and behavioral disorders, and external causes may be associated with excessive alcohol consumption on the weekends. Further research using data on individual levels of alcohol consumption are warranted.

Cardiovascular diseases (CVD) are recognized as the main cause of death worldwide. More than 17 million people died from CVD in 2008 alone accounting for 30% of worldwide deaths (1). Similar to other industrialized countries,>50% of deaths in Russia are attributed to CVD (2). However, while in neighboring Norway, the mortality from CVD has been decreasing for the past 30 years reaching 275 and 163 per 100,000 for men and women, respectively, in 2008. The trend is the opposite in Russia where the mortality from CVD increased from 509.9 in 1985 to 815.8 in 2008 for men and from 745.7 to 852.4 for women (3,4).

While elevated blood pressure, dyslipidemia, obesity, physical inactivity, insulin resistance, and smoking have been recognized as traditional risk factors for CVD, there is no consensus concerning alcohol as a risk factor for CVD (5). It is believed that the pattern of alcohol consumption defines whether its effect is cardioprotective or deleterious (6). Consumption of large amounts of alcohol during one occasion has been reported to increase the risk of coronary heart disease (7), myocardial dysfunction, fatal arrhythmias (8,9), and stroke (10). This pattern of alcohol consumption, often referred to as binge drinking (6), is typical in Russia and it has been suggested that it is a contributing factor to exceptionally high CVD mortality despite the low prevalence of the traditional risk factors among Russians (11–13). Moreover, excessive alcohol consumption can lead to premature death from poisoning by alcohol or its surrogates (14), suicide (15,16), accidents (17), and violence (18) partly explaining high mortality from external causes of death in Russia.

As in other industrialized countries, CVD are the main killers in the Arkhangelsk region with an incidence of 829 deaths per 100,000 in 2010 (19). In general, the pattern of mortality from CVD in the Arkhangelsk region is similar to that in Russia overall (20,21).

The Arkhangelsk study has shown that a considerable proportion of Russians have elevated γ-glutamyltransferase, which is a biological marker of excessive alcohol consumption (22). Moreover, using autopsy records, it has been shown that a high proportion of subjects who prematurely died from CVD consumed alcohol before death (23). Although several studies have shown associations between hazardous alcohol consumption and CVD mortality in men, in Arkhangelsk, similar associations have also been observed for women (24).

Binge drinking in Russia and the former Soviet Union is more common on weekends (25,26); therefore, the detection of elevated levels of mortality on the weekends may be considered an indirect indicator of the role of alcohol. Two studies addressed daily variations in mortality in the former Soviet Union: one in Moscow in 1993–1995 (27) and another in Lithuania in 1988–1997 (28). Both studies have shown that the proportion of deaths from CVD, accidents, violence, and alcohol poisoning is higher during weekends and Mondays than during the rest of the week. The authors explained these results by binge drinking over the weekends. However, these studies (27,28) were conducted in the 1990s. During the past decade, the economic situation in Russia has considerably improved, gross domestic product increased from 2,670 US$ in 1995 to 8,615 US$ in 2009 (29) and life expectancy increased from 64.5 in 1995 to 68.7 years in 2009 (30). Therefore, one might expect changes in the pattern of alcohol consumption in Russia, which could be reflected by changes in the pattern of deaths, which are potentially associated with alcohol. However, the only study on mortality from CVD from 2000 to 2009 has failed to demonstrate significant weekly variability of deaths. However, the number of deaths in that study was small and therefore insufficient in finding statistically significant variations in mortality by the day of the week (31).

Ambulance calls are likely to be more sensitive indicator in comparison with mortality rates particularly due to a greater number of calls compared to the number of deaths. Thus, an analysis of daily variations in ambulance calls may contribute to a better understanding of the potential impact of binge drinking on the occurrence of acute health conditions.

The aim of the study is to assess daily variations in ambulance calls for CVDs, mental and behavioral disorders, and external causes in Arkhangelsk in 2000–2008.

## Material and methods

The study was conducted in the city of Arkhangelsk, Northwest Russia, with a population of 354,000 in 2008, and representative for Russia with regard to age-and-gender distributions (20,21). Data on all ambulance calls from 1 January 2000 to 31 December 2008 were obtained from the municipal ambulance station. Only “served” ambulance calls were included, that is, those, where a team led by a medical doctor went to the patient, set a diagnosis and provided treatment. Information about patient's gender, age, doctor's diagnosis, and the date of call were recorded. Diagnoses set at each ambulance call were coded according to *International Classification of Diseases*, 10th revision. Altogether, there were 1,018,217 ambulance calls during the study period.

The disease categories selected for the analysis included CVDs (I00-99), mental and behavioral disorders (F00-F99) and external causes (S00-T98, V01-Y98) – all of which were associated with excessive alcohol consumption in previous studies (7,10,11,15–18,22–24,27,28). Absolute numbers of ambulance calls for these disease categories are presented in Table I.

**Table I:** Number of ambulance calls for CVDs, mental and behavioral disorders, external causes by age groups and gender in Arkhangelsk, 2000–2008

	18–59		60+		Total	
			
	Female	Male	Female	Male	Female	Male
CVDs (I00-99)	48475	31870	140198	42652	188673	74522
Mental and behavioral disorders (F00-F99)	17538	30746	4785	3665	22323	34411
External causes (S00-T98, V01-Y98)	34450	73207	14280	7722	48730	80929
Total	100463	135823	159263	54039	259726	189862

To ensure comparability of the results of this study with findings from other studies, proportions of ambulance calls by weekdays were calculated separately by gender, age groups (18–59, and 60+years) for all disease categories. Altogether, 26,288 (5.48%) calls to children and 3,642 (0.76%) calls where age was not registered were excluded from the analysis.

Statistical analysis was performed using SPSS Statistics, version 18.0 (SPSS Inc., Chicago, IL). For the assessment of daily variation in ambulance calls, Pearson's Chi-squared tests were used. Second, given that binge drinking is expected to have a delayed effect, the ratio of the number of calls not only on weekends but also on Monday versus the rest of the week was calculated as in other studies (28,31,32). In addition, the correction for the number of days of the week was performed:

$$\text{Ratio=}\frac{{\text{Calls}}_{\text{Sa–Mo}}}{{\text{Calls}}_{\text{Tu–Fr}}*0.75}$$

Ratios were presented with 95% confidence intervals (CIs) calculated using WinPepi software, version 4.55. If the CIs did not include 1, the difference in the number of calls on weekends plus Mondays compared to the rest of the week was considered statistically significant.

No permission from the ethical committee is required according to Russian regulations because the database contained only aggregated depersonalized data.

## Results

Tables II and III present daily variations in ambulance calls for CVD, mental and behavioral disorders and external causes by age groups and sex. The results of the supplementary analyses with the number of calls on weekends and Mondays versus the rest of the week are also presented in Tables II and III.

**Table II:** Daily variation in ambulance calls for CVDs, mental and behavioral disorders, and external causes at the age 18–59 in Arkhangelsk, 2000–2008

	CVDs (I00-99)	Mental and behavioral disorders (F00-F99)	External causes (S00-T98, V01-Y98)
	
	n (%)	n (%)	n (%)
Women			
Monday	7118 (14.7)	2520 (14.4)	4959 (14.4)
Tuesday	6913 (14.3)	2432 (13.9)	4682 (13.6)
Wednesday	7082 (14.6)	2414 (13.8)	4608 (13.4)
Thursday	6750 (13.9)	2462 (14.0)	4507 (13.1)
Friday	6709 (13.8)	2509 (14.3)	4713 (13.7)
Saturday	6802 (14.0)	2535 (14.5)	5406 (15.7)
Sunday	7101 (14.7)	2666 (15.2)	5575 (16.2)
p value for χ^2^ (df=6)	<0.001	0.009	<0.001
Sa–Mo	21021	7721	15940
Tu–Fr	27454	9817	18510
Ratio_Sa–Mo/Tu–Fr/0.75_ (95% CI)	1.02 (1.00–1.04)	1.05 (1.02–1.08)	1.15 (1.12–1.17)
Men			
Monday	4721 (14.8)	4511 (14.7)	10209 (14.0)
Tuesday	4585 (14.4)	4405 (14.3)	9740 (13.3)
Wednesday	4621 (14.5)	4466 (14.5)	9515 (13.0)
Thursday	4378 (13.7)	4249 (13.8)	9636 (13.2)
Friday	4372 (13.7)	4288 (13.9)	10078 (13.8)
Saturday	4521 (14.2)	4448 (14.5)	12073 (16.5)
Sunday	4672 (14.7)	4379 (14.2)	11956 (16.3)
p value for χ^2^ (df=6)	<0.001	0.054	<0.001
Sa–Mo	13914	13338	34238
Tu–Fr	17956	17408	38969
Ratio_Sa–Mo/Tu–Fr/0.75_ (95% CI)	1.03 (1.01–1.06)	1.02 (1.00–1.05)	1.17 (1.15–1.19)

**Table III:** Daily variation in ambulance calls for CVDs, mental and behavioral disorders, and external causes at the age 60 and older in Arkhangelsk, 2000–2008

	CVDs (I00-99)	Mental and behavioral disorders (F00-F99)	External causes (S00-T98, V01-Y98)
	
	n (%)	n (%)	n (%)
Women			
Monday	20079 (14.3)	703 (14.7)	2105 (14.7)
Tuesday	19848 (14.2)	660 (13.8)	2096 (14.7)
Wednesday	19975 (14.3)	645 (13.5)	1989 (13.9)
Thursday	19767 (14.1)	769 (16.1)	1941 (13.6)
Friday	19479 (13.9)	627 (13.1)	1982 (13.9)
Saturday	20527 (14.6)	692 (14.5)	2087 (14.6)
Sunday	20523 (14.6)	689 (14.4)	2080 (14.6)
p value for χ^2^ (df = 6)	<0.001	0.004	0.040
Sa–Mo	61129	2084	6272
Tu–Fr	79069	2701	8008
Ratio_Sa–Mo/Tu–Fr/0.75_ (95% CI)	1.03 (1.02–1.04)	1.03 (0.97–1.09)	1.04 (1.01–1.08)
Men			
Monday	6194 (14.5)	510 (13.9)	1137 (14.7)
Tuesday	6046 (14.2)	522 (14.2)	1167 (15.1)
Wednesday	6080 (14.3)	559 (15.3)	1102 (14.3)
Thursday	6105 (14.3)	488 (13.3)	1034 (13.4)
Friday	5956 (14.0)	495 (13.5)	999 (12.9)
Saturday	6013 (14.1)	550 (15.0)	1158 (15.0)
Sunday	6258 (14.7)	541 (14.8)	1125 (14.6)
p value for χ^2^ (df=6)	0.098	0.195	0.001
Sa–Mo	18465	1601	3420
Tu–Fr	24187	2064	4302
Ratio_Sa–Mo/Tu–Fr/0.75_ (95% CI)	1.02 (1.00–1.04)	1.03 (0.96–1.11)	1.06 (1.01–1.11)

Calls for CVD followed a weekly pattern with calls peaking on Sundays and Mondays. This was statistically significant for women of both age groups and for men aged 18–59 years. For men 60 years and older, a higher proportion of calls for CVD was also observed during Sundays and Mondays. Ratios also showed a greater number of calls for CVD during Saturdays, Sundays, and Mondays for both age and gender groups, varying between 2 and 3% excess calls.

Daily variation in ambulance calls for mental and behavioral disorders with greater number of calls on weekends and Mondays was found both in women and men aged 18–59 years.

Calls for external causes for both men and women were more common on weekends and Mondays than during other days in both age groups. Ratios showed more ambulance calls for external causes during Saturdays, Sundays, and Mondays, varying between 4 and 17% excess calls.

## Discussion

We observed significant variation in ambulance calls for CVDs, mental and behavioral disorders and external causes in Arkhangelsk. Similar to the results of other studies, we speculate that the elevated number of calls on weekends and Mondays can partially be explained by the excessive alcohol consumption on the weekends.

The main strength of our study is that all served ambulance calls to the Arkhangelsk ambulance station were included during 2000–2008. There in only one ambulance station in the city and by including all calls allowed avoidance of selection bias thus minimizing the risk of random error. Moreover, the large sample size provided an opportunity to assess even small variations in the number of ambulance calls. Nevertheless, the results should be interpreted cautiously taking into consideration potential limitations of the study. The medical doctor who served the ambulance call and visited the patient set diagnoses, which were used in this study. A correct diagnosis is difficult to set without biochemical, instrumental, and other methods, leaving chance for misclassification bias, although this bias is unlikely to vary across the week and thus can be considered as non-differential. Another limitation of the study is lack of data about patterns of individual alcohol consumption. At the same time, the daily variation in calls related to causes more directly related to alcohol could be considered as a proxy measure of excessive drinking on weekends. However, given the complexity of ICD-10 codes for mental and behavioral disorders due to the use of alcohol and the toxic effect of alcohol, these subcategories were not analyzed separately. Furthermore, it should be noted that drinking patterns may differ within such a large age group (18–59), but detailed analyzes in smaller age groups will be underpowered to find any associations and complicate comparisons with previous studies. In addition, we used aggregated data; therefore, ecological bias cannot be ruled out. Moreover, given the nature of the data it is not possible to identify those who called the ambulance more than once and assess whether multiple calls could affect the estimates.

Direct comparisons of our results with the results from other studies are difficult, because no studies on daily variations of ambulance calls were published from Russia. However, a few studies on variations in mortality were published during the 1990s. In the Lithuanian study, the mortality for selected causes were analyzed for 20–59 years old adults (28). In both women and men, the peak of mortality from CVD was observed on Mondays (15.7 and 15.6%, respectively). Our results also showed that the peak of calls for CVD was registered on Sundays and Mondays in women (14.7%) and on Mondays in men (14.8%) in the age group 18–59 years.

In Lithuania, the greater numbers of deaths from accidents and violence were reported on weekends (15.0% in women on Sundays and 15.1% in men on Saturdays) (28). According to the results of our study, the peak of ambulance calls for external causes had a similar pattern (16.2% in women on Sundays and 16.5% in men on Saturdays).

Similar to our results, the study conducted in Moscow also revealed a greater number of deaths from accidents and violence during weekends. Likewise, a higher number of deaths from CVD on Saturdays, Sundays, and Mondays was also observed than during the rest of the week (27).

The authors of both Lithuanian and Moscow studies explain these findings by the potential impact of binge drinking, although individual alcohol consumption was not analyzed as in our study.

Previous studies have shown that alcohol is one of the major risk factors for mortality in Russia, especially for men of working age (33). Based on this, we can assume that the younger male working population will be more likely to consume alcohol on weekends. Given some delayed effects of alcohol, we also include Mondays in our analysis of weekends. Our results do not contradict this hypothesis: for men aged 18–59 years, the peak of calls for nearly all selected causes was observed on weekends and Mondays. Moreover, it is believed that vodka and other liquors are the most common drinks consumed in Russia (34,35). It has been shown that an increase in vodka sales by 1 L increases mortality from CVD and suicide rates by 5.3 and 9.3% in men and by 3.7 and 6% in women, respectively (16,36). According to the results of our study, more calls were registered on Sundays and Mondays for CVD and on weekends for external causes among the 18–59 year-olds. In addition, along with the overall volume of alcohol consumer, the pattern of drinking is also important. The Arkhangelsk population-based study has reported that 52.3% of men and 17.3% of women had hazardous patterns of alcohol consumption (≥80 g of pure alcohol at least one time per month on one occasion) (22). Elevated levels of γ-glutamyltransferase, which is a specific biological marker of alcohol intake, was more than two times higher in the Arkhangelsk population than in similar studies (22). These data also may indirectly support the hypothesis about the excessive drinking on the weekends being related to the greater number of calls for the above-mentioned causes. It should be noted that this pattern was also observed for people aged 60 years and older for CVD and external causes.

To assess individual-level data on the role of alcohol in the Russian mortality crisis, several studies based on autopsy reports were performed (23,37). The Barnaul study found that 49% of men and 43% of women aged 35–69 years, who have died from circulatory diseases, had alcohol detected in the blood. Also, alcohol was detected in 76% of men and 65% of women of that age who have died from external causes (37). The Arkhangelsk study has also found that about one-third of men and women under 60 years, who have died from CVD, consumed alcohol before death (23). Given these facts and assuming that binge drinking most likely happening on the weekends, our hypothesis about excessive alcohol consumption behind our results seems the most probable. However, the impact of other exposures, such as smoking or drugs use cannot be excluded.

Further population-based studies using individual data including the data on alcohol consumption are warranted to improve the understanding of the impact of excessive alcohol consumption on the occurrence of acute health conditions.

## Conclusion

Daily variations in ambulance calls for CVDs, mental and behavioral disorders, and external causes in Arkhangelsk in 2000–2008 with an increased number of calls on weekends and Mondays were observed in the age group of 18–59 years for both genders. Moreover, for those who were 60 years and older, a similar pattern was observed for CVDs and external causes. Assuming that binge drinking is common on weekends, we hypothesize that excessive alcohol consumption may at least partly explain the observed variation in calls for the studied causes.
